# Evaluation of malignant effusions using MR-based T1 mapping

**DOI:** 10.1038/s41598-021-86632-1

**Published:** 2021-03-29

**Authors:** D. Kuetting, J. Luetkens, A. Faron, A. Isaak, U. Attenberger, C. C. Pieper, L. Meffert, C. Jansen, A. Sprinkart, F. Kütting

**Affiliations:** 1 Department of Diagnostic and Interventional Radiology, University of Bonn, Venusberg Campus 1, 53105 Bonn, Germany; 2 Department of Internal Medicine III; Center of Integrated Oncology (CIO) Cologne-Bonn, University of Bonn, Bonn, Germany; 3 Department of Internal Medicine I, University of Bonn, Bonn, Germany; 4 Clinic for Gastroenterology and Hepatology, University of Cologne, Cologne, Germany

**Keywords:** Cancer imaging, Gastrointestinal cancer, Metastasis

## Abstract

Our aim was to investigate the diagnostic yield of rapid T1-mapping for the differentiation of malignant and non-malignant effusions in an ex-vivo set up. T1-mapping was performed with a fast modified Look-Locker inversion-recovery (MOLLI) acquisition and a combined turbo spin-echo and inversion-recovery sequence (TMIX) as reference. A total of 13 titrated albumin-solutions as well as 48 samples (29 ascites/pleural effusions from patients with malignancy; 19 from patients without malignancy) were examined. Samples were classified as malignant-positive histology, malignant-negative histology and non-malignant negative histology. In phantom analysis both mapping techniques correlated with albumin-content (MOLLI: r = − 0.97, TMIX: r = − 0.98). MOLLI T1 relaxation times were shorter in malignancy-positive histology fluids (2237 ± 137 ms) than in malignancy-negative histology fluids (2423 ± 357 ms) as well as than in non-malignant-negative histology fluids (2651 ± 139 ms); post hoc test for all intergroup comparisons: < 0.05. ROC analysis for differentiation between malignant and non-malignant effusions (malignant positive histology vs. all other) showed an (AUC) of 0.89 (95% CI 0.77–0.96). T1 mapping allows for non-invasive differentiation of malignant and non-malignant effusions in an ex-vivo set up.

## Introduction

Ascites and pleural effusions present a challenging diagnostic problem for clinicians in the context of suspected malignancy. Definite discrimination between malignant and benign effusions remains difficult without obtaining histological or cytological confirmation, a process that is often burdening for patients, or is missed due to inconspicuous results in initial work up. Even after obtaining a sample, microscopic analysis and cytology for malignancy yield high specificities, however false negative results are obtained in up to 40–60% of cases^1,2^. Furthermore, the volume of peritoneal/pleural fluid may be too low to allow for safe sampling. Peritoneal/pleural biopsy are validated alternatives^3,4^, however, due to their invasive nature application is limited in clinical practice.

Thus, clinically applicable non-invasive techniques need to be developed for the differentiation of malignant and benign effusions in order to ameliorate the process for patients and diagnosticians alike. In both malignant pleural effusions and ascites protein content is typically increased, as a result of impaired lymphatic drainage and increased vascular permeability. In magnetic resonance imaging T1 relaxation time in fluids is typically very long^5^ while the presence of protein reduces T1 relaxation time in pleural effusions/ascites^6,7^. The results of earlier studies indicate that T1-mapping may serve as a surrogate marker for the detection of malignant effusions^7^. Furthermore, modern T1-mapping techniques allow for rapid imaging-based appraisal of T1 relaxation times, thus principally enabling clinical application.

## Methods and materials

In order to evaluate rapid MRI T1-mapping for the discrimination of malignant/non-malignant ascites, phantom analysis (albumin solutions) and ex-vivo tests were performed.

### Study population

Samples of pleural effusions and ascites from 48 patients were examined with MRI between 04/2019 and 05/2020. Samples were drawn from 29 patients with malignancy (pleural effusions: n = 17; ascites: n = 12), as well as from 19 patients without malignancy (pleural effusions: n = 9; ascites: n = 10). All fluid samples were drawn during routine clinical procedures. All samples underwent laboratory testing on the day of the MRI examination to investigate the concentration of total protein, triglycerides, cholesterol, red blood cell (RBC) count, white blood cell (WBC) count, sodium -, potassium -, calcium – and chloride levels. Additional dedicated microscopic and cytology analysis was performed on the drawn samples. Samples were classified as malignancy-positive histology if samples were drawn from patients with malignancy and histologic/cytologic analysis revealed malignant cells; as malignancy-negative histology if samples were drawn from patients with malignancy but histologic/cytologic analysis did not reveal malignant cells; and as non-malignant negative histology if samples were drawn from patients without any known malignancies and histologic/cytologic analysis did not reveal malignant cells. The local institutional review board (E*thics Committee* of the Faculty of Medicine of *Bonn University)* approved this study; ex-vivo analysis of effusion samples was exempt from IRB review, a waiver for informed patient consent was approved. All experiments were performed in accordance with relevant guidelines and regulations.

### MRI protocol

Imaging was performed using a 1.5 T clinical MRI-system (Ingenia, Philips Healthcare, Best, The Netherlands). T1 Mapping was performed with a combined turbo spin-echo and inversion-recovery sequence (TMIX)^8^ and a fast modified look locker inversion recovery technique (MOLLI) as previously reported^9^. To account for long T1 relaxation times of fluids, MOLLI inversion times (TI) were adapted to cover a broad range of TIs from 121 to 3621 ms applying a 8–4 scheme, 7 s pause interval, a prepulse delay of 350 ms and a ΔTI of 500 ms in each cycle. Further imaging parameter of the MOLLI sequence were: repetition time (TR) = 1.9 ms, echo time (TE) = 0.85 ms, flip angle = 35°, slice thickness = 10 mm, acquisition matrix = 152 × 150, reconstructed voxel size = 1.18 × 1.18x10mm^3^, parallel imaging factor = 2, acquisition duration = 13 s.

The imaging parameters of the TMIX sequence were TR-spin-echo/TR-inversion recovery = 2000/4000 ms, TEs = 20/100 ms, flip angle = 90°, acquisition matrix = 120 × 120, reconstructed voxel size = 0.45 × 0.45x12mm^3^, acquisition duration = 8 min.

Examinations were performed on albumin emulsions (phantom; predefined albumin content) and on drawn samples of pleural and ascitic fluids (ex-vivo) to determine the impact of albumin content on T1-relaxation time as well as to evaluate whether a differentiation of malignant/non-malignant effusions is possible by means of T1-mapping. MR-testing of samples was performed after these had adapted to room temperature (21 °C).

### Phantom analysis

MRI evaluation was performed on a total of 13 albumin emulsions (Human Albumin, Behring, Germany) with varying concentrations of albumin (between 0 and 200 g/l) diluted in sodium chloride (combined fluid volume in all samples was 50 ml).

### Statistical analysis

Statistical analyses were performed using commercially available statistical software (Prism version 8, GraphPad, La Jolla, CA). Bland Altman analysis was performed for phantom analyses to determine the agreement between MOLLI and TMIX measurements of T1 relaxation times. Correlation between laboratory protein concentrations and T1 relaxation times was analyzed with Pearson’s correlation. Regression analysis was performed to determine which factors (triglyceride levels, protein content, RBC, WBC) were predictive of T1 relaxation times for ex-vivo analysis. Receiver operating characteristic (ROC) curves were plotted for ex-vivo data to determine the optimal T1-time threshold to differentiate malignant/non-malignant fluids. Inter-scan and intra-scan reproducibility was assessed by the intra-class correlation coefficient (ICC).

## Results

### Agreement TMIX and MOLLI

**Figure 1:**
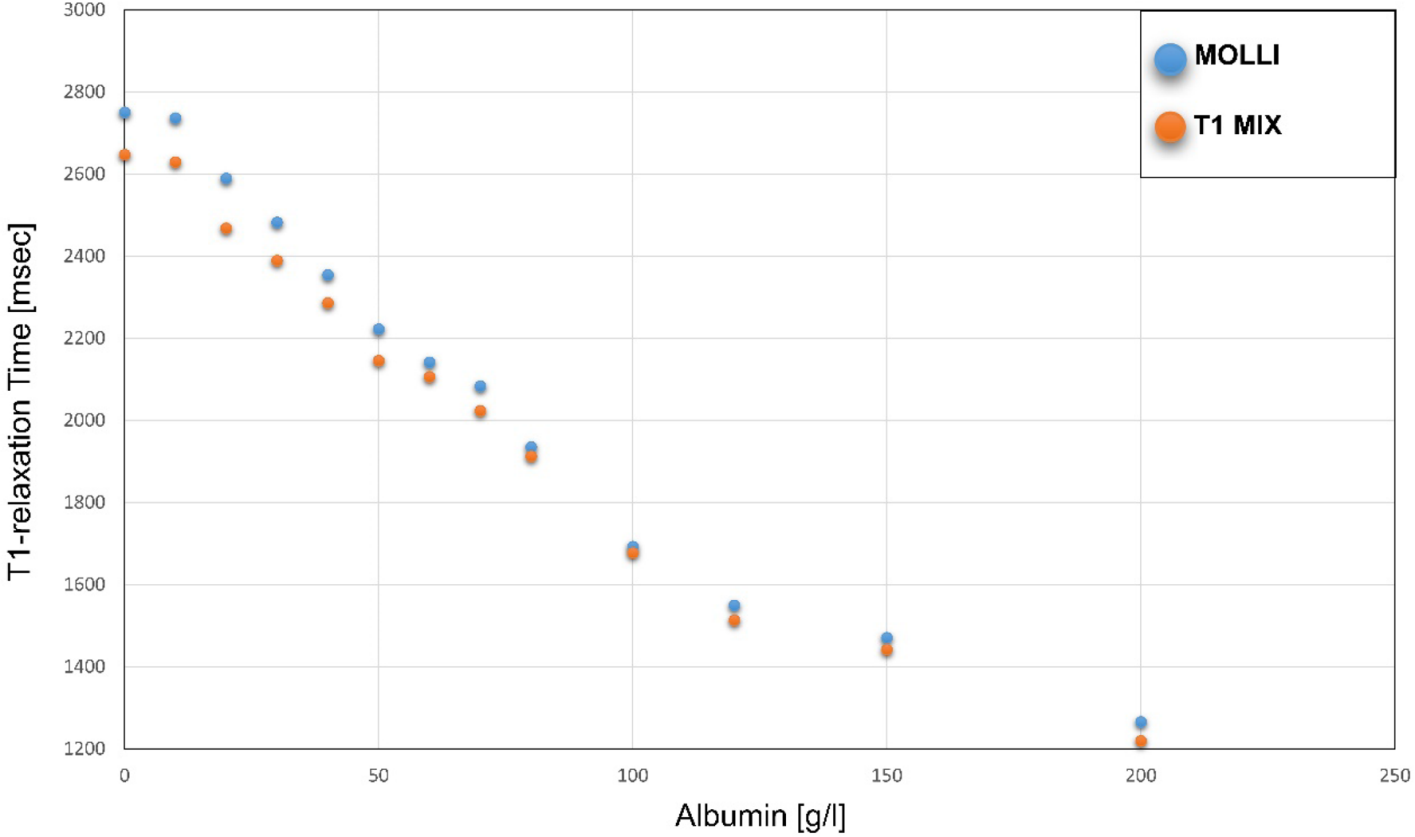
Graph displaying results of T1 relaxation time (in ms) measurement performed with MOLLI and T1-MIX on samples containing albumin (0–200 g/l) dissolved in saline.

**Figure 2:**
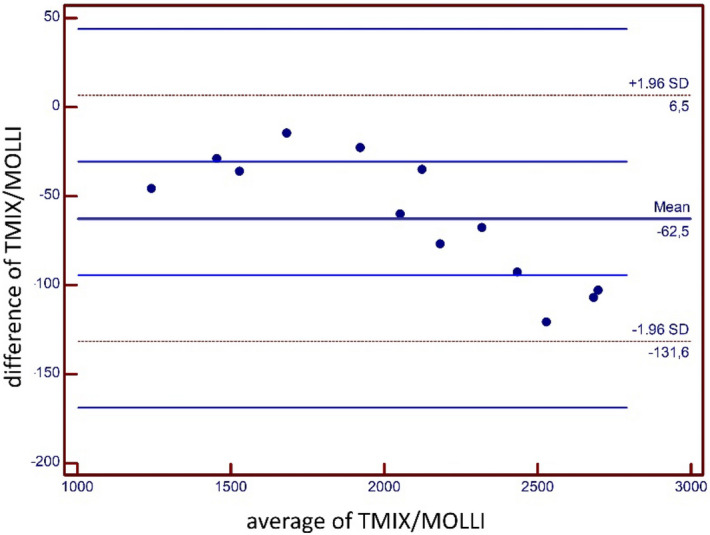
Bland–Altman plot for agreement between T1 measurements of TMIX and MOLLI for phantom analysis. Blue lines indicate 95% limits of agreement. SD = standard deviation.

Figure 1 displays TMIX and MOLLI results for T1 relaxation times of phantom analysis. Mean T1 relaxation times for phantom analysis as obtained by TMIX and MOLLI were 2035.0 ± 443.3 ms and 2097.5 ± 471.1 ms, respectively (r = 0.99; *p* < 0.001)). Bland Altman analysis revealed a strong measurement agreement between MOLLI and the reference (∆T1 = 62.5 ± 31.5 ms, 95% CI − 6.5 to 131.6 ms; Fig. 2).

Mean T1 relaxation times for ex-vivo analysis of TMIX and MOLLI were 2359.7 ± 273.8 ms and 2431.8 ± 269.4 ms, respectively. Bland Altman analysis again revealed a strong agreement of MOLLI and TMIX relaxation time measurements (∆T1 = 72.0 ± 41.8 ms, 95% CI − 9.8 to 153.9 ms).

### Phantom evaluation

Phantom analysis revealed that T1 measurements from both T1-MOLLI and TMIX measurements correlated with albumin concentrations (r = − 0.97; r = − 0.98, *p* < 0.001 each).

### Ex-vivo evaluation

**Figure 3:**
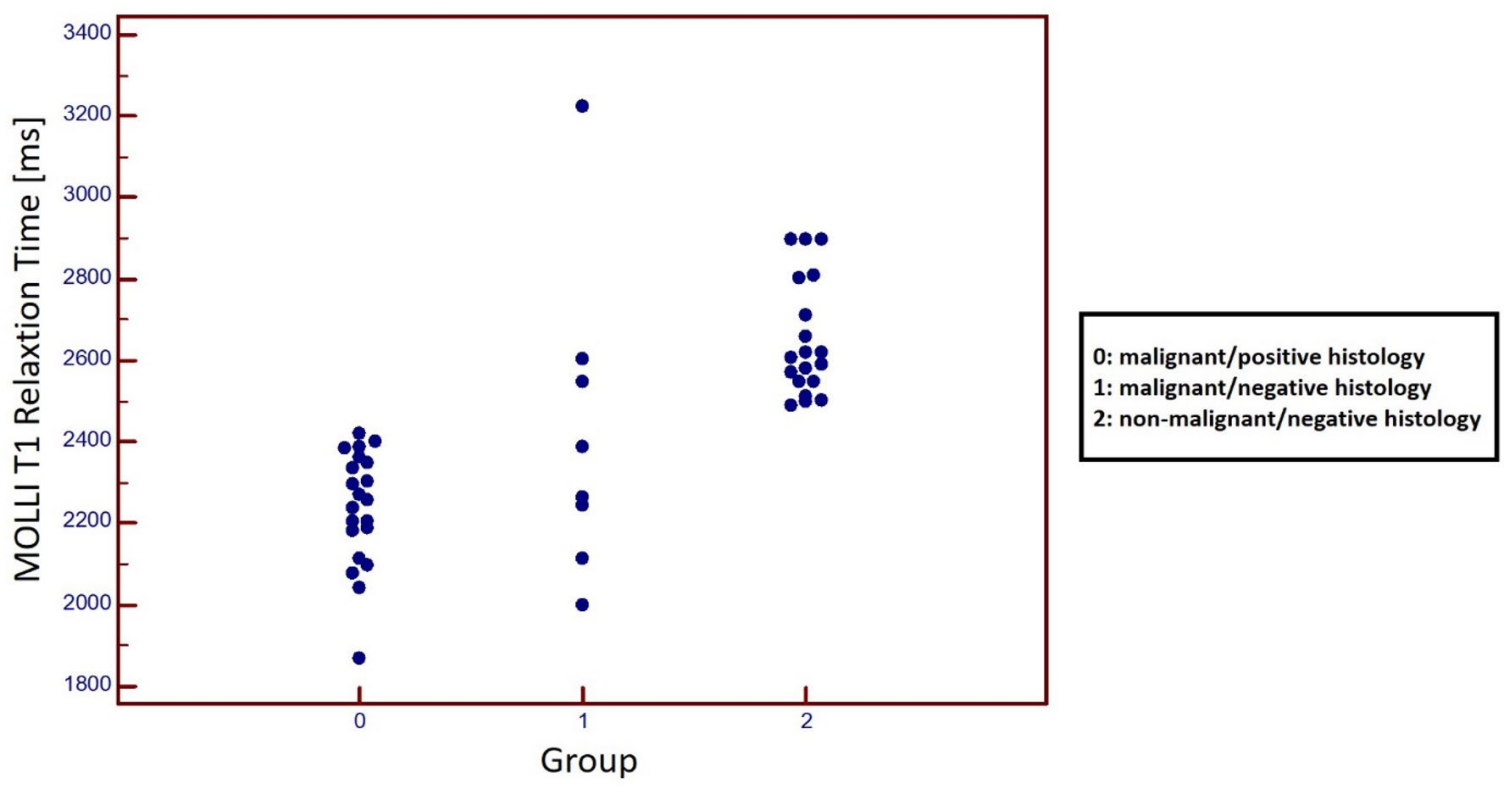
Graphs with individual plotted values show distribution of MOLLI T1 relaxation time in effusions from patients with known malignancy and positive histology/cytology (Group 0), effusions from patients with known malignancy but negative histology/cytology (Group 1) and effusions from patients without a known malignancy and negative histology/cytology (Group 2). Individual values are represented as single colored dots. One-way ANOVA for intergroup comparison showed T1 relaxation times differed between all groups (Group 0: 2237.1 ± 136.9 ms; Group 1: 2423.3 ± 357.4 ms; Group 2: 2650.5 ± 138.5 ms) (*p* < 0.001; post hoc test for all intergroup comparisons: < 0.05).

Patient characteristics as well as details of fluid composition of ex-vivo analysis are given in Table 1**.** MOLLI T1 relaxation times of malignant-positive histology fluids ranged from 1867 to 2419 ms, in malignant-negative histology from 1999 to 3223 ms and in non-malignant-negative histology from 2489 to 2897 ms; one-way ANOVA for intergroup comparison: F(2.45) = 36.1; *p* < 0.001; post hoc test for all intergroup comparisons: < 0.05; Fig. 3. Also, MOLLI T1 relaxation times were shorter when comparing histologically proven malignant fluids (2237.1 ± 136.9 ms) and all other fluids (2583.1 ± 249.2 ms, *p* < 0.001).

**Table 1:** Overview of tumor etiology (Tumor) and location (Loc) of effusions (P: pleural effusion; A: ascites) from ex-vivo studies; Effusions were classified as malignant (Histo:1) if histology or cytology was positive; otherwise effusions were classified as non-malignant (Histo:0).

PAT ID	Gr	Tumor	Loc	Hist	Nr. of Ass	Protein (g/l)	WBC (µL^−1^)	RBC (µL^−1^)	Trig. (mg/dl)	Chol. (mg/dl)	MOLLI (ms)	T1 MIX (ms)
1	0	Ovarian	A	1	3	22.6	2103	3000	16	30	2384	2295
2	0	Ovarian	A	1	3	17.6	2416	< 1000	15	28	2362	2381
3	0	Ovarian	P	1	2	19.6	2674	2000	25	32	2336	2321
4	0	Ovarian	P	1	2	18.3	2545	1500	20	30	2349	2351
5	0	Ovarian	A	1	2	33.3	5	< 1000	18	30	2187	2112
6	0	Lung	P	1	2	24.9	141	26,000	24	34	2097	1976
7	0	Ovarian	A	1	1	54.4	357	4000	261	222	1867	1836
8	0	Bladder	A	1	1	41	3181	16,000	38	88	2237	2137
9	0	Pancreas	A	1	2	26.5	104	< 1000	27	34	2295	2223
10	0	Pancreas	P	1	2	28.5	106	< 1000	53	15	2115	2045
11	0	Pancreas	P	1	2	27.5	105	< 1000	40	24,5	2205	2134
12	0	Pancreas	P	1	1	37	410	7000	29	42	2183	2045
13	0	Lymphoma	P	1	1	29.3	874	2000	351	99	2205	2179
14	0	Lymphoma	A	1	1	30	500	10,000	133	69	2419	2330
15	0	Lymphoma	P	1	1	29.3	874	2000	351	99	2302	2223
16	0	Sarcoma	P	1	2	32	233	2000	30	75	2042	2003
17	0	Breast	P	1	2	19	38	< 1000	13	34	2269	2197
18	0	Breast	P	1	3	19	20	< 1000	15	32	2257	2172
19	0	Lung	P	1	1	25	173	8000	16	30	2079	2003
20	0	Pancreas	A	1	2	29	54	1000	21	29	2389	2305
21	0	Breast	P	1	2	24	121	4000	28	24	2401	2368
22	1	HCC	A	0	1	12	513	56,000	30	21	1999	1951
23	1	Pancreas	A	0	2	13.4	500	10,000	26	11	2389	2319
24	1	CCC	A	0	1	23.2	962	< 1000	26	26	2265	2157
25	1	Breast	P	0	1	28.5	2129	17,000	15	28	2114	2044
26	1	Pancreas	A	0	1	24.2	1072	214,000	39	26	2244	2142
27	1	Lung	P	0	2	10.6	3019	4000	12	13	2604	2602
28	1	Pancreas	P	0	2	16	2436	7000	15	103	3223	3260
29	1	Lymphoma	P	0	1	11.4	73	< 1000	31	20	2548	2452
30	2	–	A	0	1	9.7	164	< 1000	28	12	2621	2501
31	2	–	A	0	1	12.1	62	< 1000	18	12	2897	2847
32	2	–	P	0	1	2.7	212	< 1000	9	4	2618	2597
33	2	–	A	0	1	3.7	71	< 1000	9	7	2580	2540
34	2	–	A	0	1	9.6	358	< 1000	44	16	2810	2690
35	2	–	P	0	1	9	25	< 1000	43	13	2897	2861
36	2	–	A	0	1	19	135	< 1000	49	17	2710	2613
37	2	–	P	0	1	16	87	5000	50	10	2897	2799
38	2	–	A	0	1	12	201	< 1000	64	9	2803	2654
39	2	–	P	0	1	5.9	46	< 1000	13	5	2591	2493
40	2	–	P	0	1	5.7	40	< 1000	14	5	2659	2577
41	2	–	A	0	1	11.4	73	< 1000	31	20	2548	2452
42	2	–	A	0	1	10.2	103	< 1000	21	13	2512	2392
43	2	–	A	0	1	9.4	154	3000	28	12	2503	2435
44	2	–	P	0	1	5.1	45	< 1000	15	8	2605	2535
45	2	–	A	0	1	11.7	68	< 1000	32	22	2548	2396
46	2	–	P	0	1	10.8	99	< 1000	10	15	2499	2392
47	2	–	P	0	1	9.2	161	2600	26	13	2572	2481
48	2	–	P	0	1	9.4	46	< 1000	27	16	2489	2449

Number of histological/cytologic assessments performed (Nr. of Ass.). Laboratory testing was performed to determine levels of protein, white blood cells (WBC), red blood cells (RBC), triglycerides (trig.), cholesterol (chol.). MRI testing war performed to determine T1-relaxation time with MOLLI and T1-MIX.

**Figure 4:**
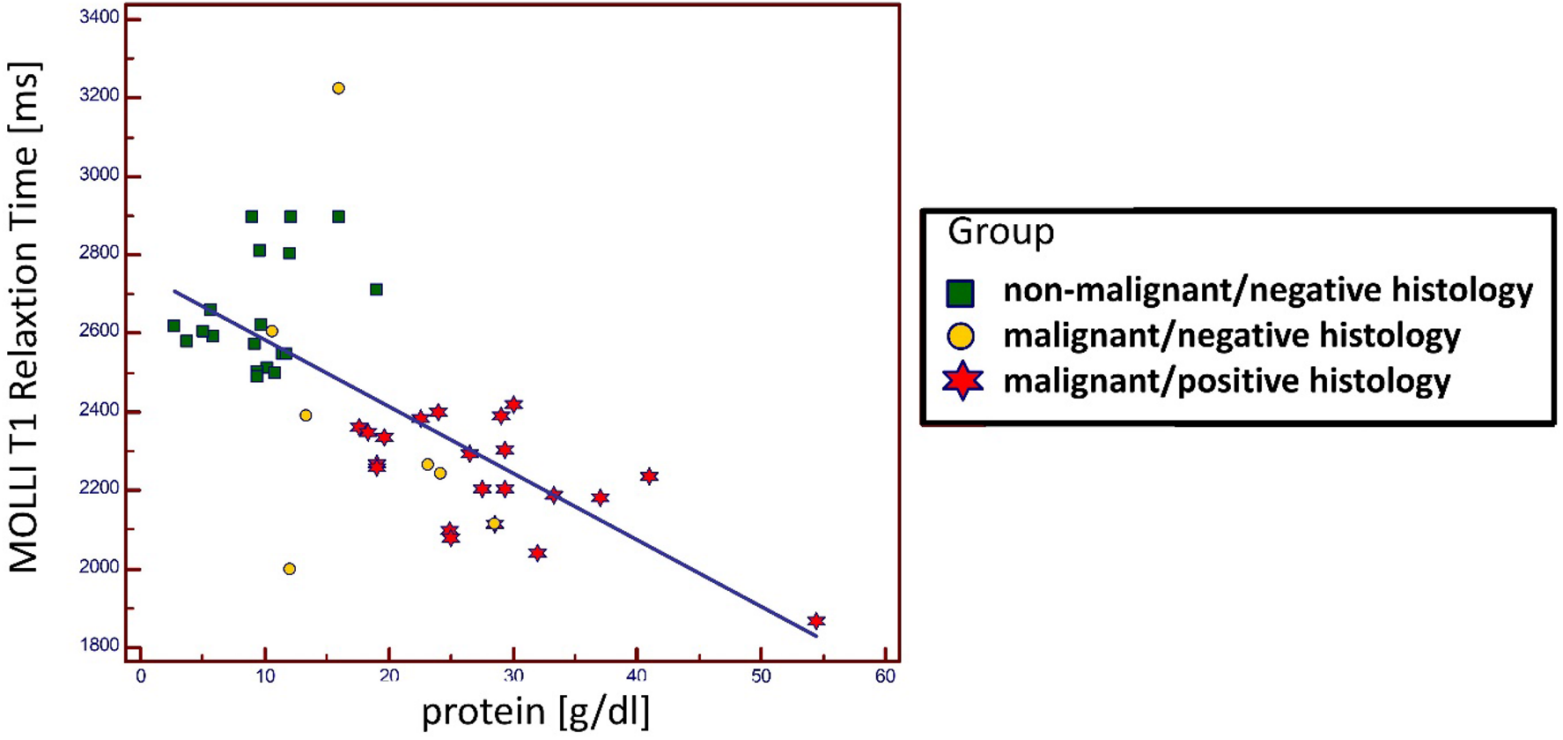
Regression analysis for ex-vivo MOLLI T1 Relaxation time measurements: MOLLI T1-Relaxation time (dependent variable) and protein content (independent variable). Linear regression established that for ex-vivo measurements, protein content was associated with MOLLI T1-relaxation time measurements (r = − 0.69) Effusions were defined as non-malignant/negative histology (green squares) if patients had no known malignancy and histology/cytology was negative; as malignant/negative histology (red star) if patients had known malignancy but histology/cytology was negative; as malignant/positive histology (yellow circle) if patients had known malignancy and histology/cytology was positive.

Linear regression established an association between protein content and MOLLI T1-measurements in our ex-vivo setup. (T1 [ms] = 2753.7—16.9 · x [g/l], *p* < 0.001, r = − 0.69) (Fig. 4). Triglyceride content (r = − 0.24; *p* = 0.09), cholesterol (r = 0.19; *p* = 0.23), RBC count (r = − 0.19; *p* = 0.18), WBC count (r = 0.24; *p* = 0.13), calcium (r = 0.14; *p* = 0.37), potassium (r = − 0.03; *p* = 0.99), sodium (r = 0.05; *p* = 0.77), phosphate (r = − 0.2; *p* = 0.18) were not associated with MOLLI T1-measurement variability.

**Figure 5:**
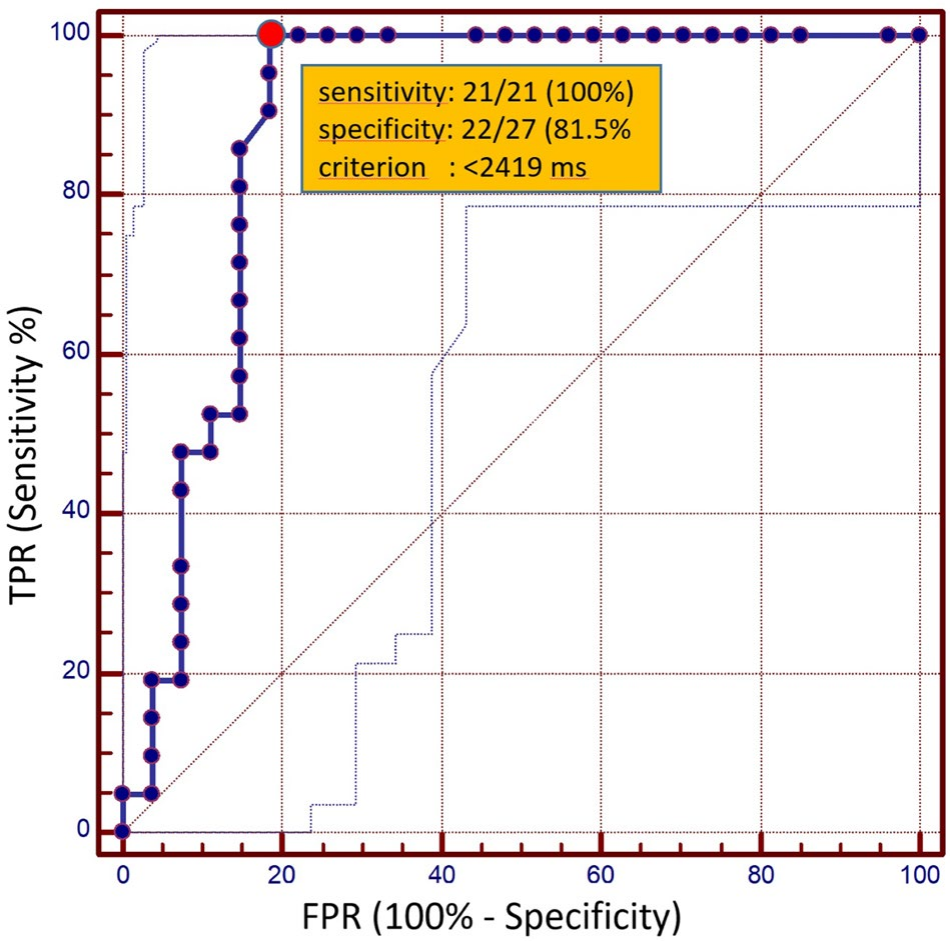
ROC curves. Receiver operating characteristic (ROC) curves for MOLLI T1 relaxation time based differentiation of malignant and non-malignant effusions from ex vivo analysis (AUC: 0.89). The red dot indicates the best performing cut-off value (criterion: T1-relaxation time < 2419 ms) for the MOLLI T1 Mapping technique with a sensitivity of 100% (95% CI 83.9–100%) and a specificity of 81.5% (95% CI 61.9–93.7%) and a corresponding AUC of 0.89 (95% CI 0.77 to 0.964) in the differentiation of malignant and non-malignant effusions. The dotted lines indicate the 95% confidence interval. TPR: true positive rate; FPR: false positive rate.

ROC analysis for differentiation between histologically proven malignant (malignant-positive histology) and non-malignant fluids (malignancy-negative histology + non-malignancy-negative histology) (Fig. 5) showed an area under the curve (AUC) of 0.893 (95% CI 0.77 to 0.964). Using a cut-off value of < 2419 ms for MOLLI T1 performed best in distinguishing malignant from non-malignant effusions with a sensitivity and specificity of 21/21 (100% [95% CI 83.9–100%]) and 22/27 (81.5% [95% CI 61.9–93.7%]), respectively, by Youden's index method.

## Discussion

Although multiple noninvasive visualization techniques have been suggested for the differentiation of malignant and non-malignant effusions^10,11^, until now no non-invasive tool has been clinically implemented.

As a direct visualization of tumor cells is not possible with imaging techniques currently available, a reliable surrogate marker is necessary to indicate whether an effusion is malignant or not. Apart from cancerous and inflammatory cells, malignant effusions typically include protein^12^. Protein content is a significant determinant of T1 relaxation time, especially in fluids where relaxation times are otherwise very long^13^. T1 Mapping has previously been investigated in earlier studies dating back to the 80 s and 90 s^6,7^. However, ordinary T1 Mapping is extremely time-consuming, especially when a large field of view is employed.

MOLLI T1 Mapping could therefore potentially serve as a clinical applicable tool for the evaluation of effusions, as acquisition is rapid and results remain robust^9^. Until now MOLLI has only been validated for cardiac and hepatic T1 Mapping, thus we initially performed phantom analysis in fluid solutions where we found good agreement with a validated reference for T1 Mapping^14^ and strong correlation with albumin content (r = − 0.97, *p* < 0.01).

As a next step, we investigated the diagnostic significance of rapid T1-Mapping for the differentiation between malignant and non-malignant effusions. Ex-vivo regression analysis showed that apart from protein content, none of the other investigated factors were associated with T1-mapping results. These results are in line with previous data that suggest T1-relaxation rates (1/T1) of ascites are linearly proportional to protein content in non-malignant cases^7^. In contrast to non-malignant ascites, where paramagnetic ions are only marginally present^15^, higher concentrations of iron can be found in malignant ascites due to secondary iron overload. As the paramagnetic contribution of iron increases relaxation^7^, this phenomenon is expected to increase the ability to separate malignant and non-malignant ascites by means of T1-mapping.

Results derived from ex-vivo analysis revealed that T1-mapping allowed for consistent detection of malignant effusions (defined as microscopic/cytologic confirmation of malignancy) with a high sensitivity/specificity. ROC analysis demonstrated that using a T1-Mapping cut-off value of < 2419 ms, yielded a sensitivity of 21/21 (100% [95% CI 83.9–100%]) and a specificity 22/27 (81.5% [95% CI 61.9–93.7%]) with a corresponding AUC of 0.97 (95% CI 0.8 to 1.0) in the differentiation of chylous and non-chylous effusions.

As shown in Figs. 3 and 4, T1-relaxation times differed considerably in patients with proven malignant effusion and patients without malignancy, while results partially overlapped in patients with malignancy/positive histology (in effusion) and patients with malignancy/negative histology (in effusion). This may be explained by the composition of the various effusions. Albumin content as well as cell count were higher in effusions from patients with malignancy/negative histology than in patients without malignancy. The reason for the discrepancy between compositions remains unclear. Patients from the malignancy/negative histology group may have been false negatives in histologic assessment, as sensitivity varies greatly among different tumor types. Routine cytological evaluation of pleural fluid has a diagnostic yield ranging from 62 to 90% in patient populations with malignant pleural effusions^16^. As oncologic patients are prone to infectious complications, another likely explanation may be non-malignant effusions secondary to infections only indirectly linked to the respective tumor. These issues may be sorted out in future analyses when utilizing methods beyond cytology to invasively differentiate malignant from non-malignant effusions (i.e. GLUT-1, tumor markers etc).

Unlike TMIX, MOLLI may be used for T1-Mapping in non-stationary tissue (e.g. heart and liver), due to the much shorter acquisition duration of MOLLI T1-Mapping compared the reference sequence. This is particularly relevant, as robust mapping of pleural effusions and ascites requires fast acquisition in order to prevent breath excursions or bowel movements from falsifying results.

There are several limitations of this study. This study was conceived as a feasibility study: our patient cohort (48 study participants) was relatively small and all examinations were performed ex-vivo at 21 °C. T1-mapping has been shown to be temperature dependent^7^; thus T1 cut-off values are expected to vary in-vivo. T1 relaxation times are reduced in fluids with elevated protein content, thus apart from malignant effusions, other exudative effusions (e.g. bacterial peritonitis, pleural empyema) will typically also show increased protein content. Although infectious effusions were not included in the current study, it is to be expected these will also show reduced T1 relaxation times. Thus, in cases where an infectious cause of effusions cannot be excluded clinically, T1-mapping may be of reduced benefit. This is the first study investigating the value of rapid non-invasive T1-Mapping for the differentiation of malignant/non-malignant ascites and pleural effusions. With regard to abdominal imaging, differentiating peritoneal carcinosis from non-malignant (exsudative) diseases with increased protein concentrations such as bacterial peritonitis may remain difficult. The impact of paramagnetic ions in malignant ascites on T1-Mapping results was not additionally investigated in this study, however it has previously been shown that paramagnetic ions lead to more extensive T1-relaxation time reduction than may be expected based on protein content ^7^.

Overall, this promising preliminary data warrants further investigation within a larger cohort and in an in-vivo context in order to answer the remaining questions comprehensivelyThe results presented here indicate that MOLLI T1-Mapping may serve as a rapid non-invasive diagnostic tool for the discrimination of malignant ascites and pleural effusions. Hopefully, in the future it will help guide clinicians in this often diagnostically difficult field.
